# Identification of a Novel Papillomavirus Type (MfoiPV1) Associated with Acrochordon in a Stone Marten (*Martes foina*)

**DOI:** 10.3390/pathogens10050539

**Published:** 2021-04-30

**Authors:** Urška Kuhar, Diana Žele Vengušt, Urška Jamnikar-Ciglenečki, Gorazd Vengušt

**Affiliations:** 1 Institute of Microbiology and Parasitology, Veterinary Faculty, University of Ljubljana, Gerbičeva 60, 1000 Ljubljana, Slovenia; urska.kuhar@vf.uni-lj.si; 2 Institute of Pathology, Wild Animals, Fish and Bees, Veterinary Faculty, University of Ljubljana, Gerbičeva 60, 1000 Ljubljana, Slovenia; diana.zelevengust@vf.uni-lj.si; 3 Institute of Food Safety, Feed and Environment, Veterinary Faculty, University of Ljubljana, Gerbičeva 60, 1000 Ljubljana, Slovenia; urska.jamnikar@vf.uni-lj.si

**Keywords:** papillomavirus, dyonupapillomavirus, stone marten, *Martes foina*, NGS, MfoiPV1

## Abstract

Papillomaviruses (PVs) are an extremely large group of viruses that cause skin and mucosal infections in humans and various domestic and wild animals. Nevertheless, there is limited knowledge about PVs in wildlife hosts, including mustelid species. This study describes a case in stone marten (*Martes foina*) with a clinical manifestation of skin tumor, which is rather atypical for infections with PVs. The result of the papillomavirus PCR performed on the skin tumor sample was positive, and the complete PV genome was determined in the studied sample using next-generation sequencing technology. The analysis of the PV genome revealed infection of the stone marten with a putative new PV type belonging to the *Dyonupapillomavirus* genus. The proposed new stone marten PV type was named MfoiPV1.

## 1. Introduction

Papillomaviruses (PVs) are small, nonenveloped viruses with a circular, double-stranded DNA genome. The genome ranges in size from 5748 to 8607 bp and can be divided into three functional regions: the long control region (LCR), the early region (E) and the late region (L). The coding regions, namely the E and L regions, contain 8–10 open reading frames (ORFs) encoding proteins with regulatory function during virus replication (E1 and E2) and proteins involved in virion capsid formation (L1 and L2). The early E1 and E2 proteins and the late L1 and L2 proteins form the basis of the viral genome and are present in all sequenced papillomaviruses [1,2,3]. Four early ORFs, encoding the E4, E5, E6, and E7 proteins, are common in the mammalian clade of papillomaviruses. The E3 and E8 ORFs have additionally been identified in mammalian papillomaviruses, but typically do not encode individual proteins [4]. The LCR is located between the L1 and E6 ORFs and contains the origin of replication and transcription factor binding sites [1,2]. PVs are epiteilothropic viruses that infect skin and mucosal epithelia in humans and various animals. They can cause subclinical infections in infected individuals or cause various neoplastic changes ranging from benign, self-limited warts to cancer [5,6,7,8,9]. PVs are known to be highly host-specific [1], however, some exceptions of cross-infection have been reported [7,10,11,12].

More than 400 human and animal PV reference genomes are listed in the curated database Papillomavirus Episteme (PaVE) [13] (http://pave.niaid.nih.gov, accessed on 8 February 2021) [14,15]. Only nearly half of these genomes are of animal origin, indicating a lack of knowledge about PV diversity in animals, particularly in wildlife hosts [16,17,18]. PVs have been found not only in mammals, but also in fish [19], birds [20,21,22] and reptiles [23,24]. The most studied PVs in animals are from domestic hosts, including cattle [7,8,25], dogs [8,26], cats [8,27,28], horses [29,30], and sheep [31,32]. In addition to domestic animals, PVs have also been studied in various wildlife hosts, such as the ocelot [33], bottlenose dolphin [34], gray wolf [35], red fox [36], European badger [37], tree shrew [38], giant panda [39], roe and red deer [40,41,42,43,44,45], etc. Considering PVs found in Carnivora hosts, 50 types are recognized in the PaVE database, with almost half of them belonging to canine hosts [8,13].

The stone marten, sometimes called “beech marten” (*Martes foina*, Erxleben, 1777), is a small carnivore species belonging to the family *Mustelidae*. It is distributed over large parts of Europe from Portugal to Eastern Russia. Among all European representatives of the marten family, the stone marten is the only species whose numbers are increasing and it is one of the most widespread mustelids in the Eurasian region [46]. The stone marten occurs throughout the territory of Slovenia, and inhabits light, open forests, rocky areas, and urban habitats [47]. The annual hunting bag in 2019 was 927 animals [48]. Despite its wide distribution, little is known about pathogens infecting this species and nothing is known about PVs in this species.

The aim of the present study was first to investigate a skin tumor case in a stone marten using histopathology and viral diagnostics by means of PCR. Subsequently, the aim of this study was to determine the nucleotide sequence of the complete PV genome by next generation sequencing (NGS) and to characterize it phylogenetically.

## 2. Results

### 2.1. Case Description

**Figure 1:**
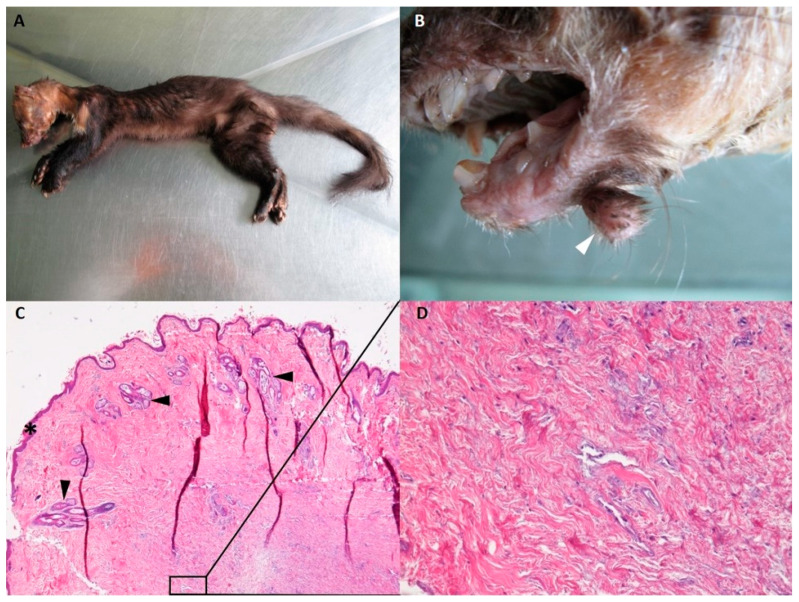
(**A**–**C**) Gross pathology and microscopic images of the acrochordon (white arrow) in a stone marten (*Martes foina*). (**C**) Microscopic presentation. An acrochordon, a fibroepithelial papilloma covered by a slightly hyperplastic and hyperkeratotic epithelium of variable thickness (*) with hair follicles (black arrows). H&E 40×. (**D**) Microscopic presentation. The dermis is composed of mature collagen fibers running in different directions. H&E 200×.

The stone marten (male, apparently two to three years old) examined was in poor body condition. Moderate decomposition was observed. The animal was severely emaciated, with serous atrophy of adipose tissue and moderate muscle wasting (Figure 1A). The condition of the teeth was very poor. Postmortem examination revealed a solitary, firm, round, hairy skin tumor measuring 4 × 3 × 3 mm^3^ located on the skin between the mandibles of the infected animal (Figure 1B).

### 2.2. Histopathology of the Skin Tumor

Histopathological examination of the skin tumor revealed elements characteristic of an acrochordon, a fibroepithelial papilloma covered by a slightly hyperplastic and hyperkeratotic epithelium of variable thickness (Figure 1C,D).

### 2.3. PCR, NGS and Genome Analysis

**Figure 2:**
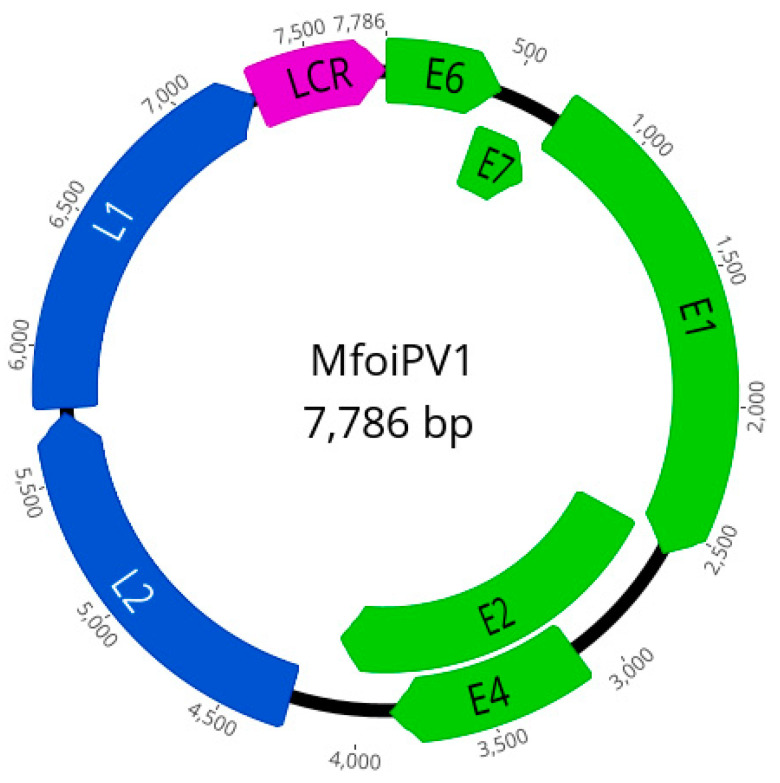
Schematic presentation of the genome organization of the novel PV type (MfoiPV1) belonging to *Dyonupapillomavirus* genus from a stone marten.

The result of papillomavirus PCR performed on the skin tumor sample was positive, so the sample was subjected to NGS to determine the complete genome sequence of *Martes foina* PV (MfoiPV1). NGS yielded 15,107,010 paired reads that were used for de novo assembly. The de novo assembled contigs were subjected to a BlastX search, which revealed a contig belonging to papillomavirus, while no contig belonging to other viruses was found. From this contig, a complete circular MfoiPV1 genome was constructed. The sequenced reads were mapped back against the assembled MfoiPV1 genome, with 625 reads mapped. The complete MfoiPV1 genome was 7786 bp long with a GC content of 58% and had a similar organization to other PV types belonging to the *Dyonupapillomavirus* genus. Seven ORFs were predicted on the MfoiPV1 genome, encoding five early (E) proteins (E6, E7, E1, E2, E4 (nested in E2)) and two late (L) proteins (L2, L1). The long control region (LCR) was located between L1 and E6 (at genome position 7274–7786, length 513 bp) and a non-coding region (at genome position 4117–4267, length 151 bp) was located between the early and late regions (Table 1, Figure 2).

The E6 protein and the E7 protein were predicted to contain two and one conserved zinc-binding domains (CX2C–X29–CX2C), respectively. The E7 protein was also predicted to contain the retinoblastoma (RB) protein binding site (LXCXE). For the E1 protein, one ATP-dependent helicase motif (GXXXXGK[TS]) and six cyclin A interaction motifs (RXL) were predicted. For the L1 protein, the bipartite nuclear localization signal (NLS) was predicted. In the LCR, the five E2 binding sites (ACCGNNNNCGGT), one SP1 binding site (GGCGGG), and one TATA-like box (TATA[AT]A[AT]) were identified. One NF1 binding site was found in the E2 gene and four polyadenylation signal sites were also found in the E1, E2, L2 and L1 genes (Table 1).

### 2.4. Phylogenetic Analysis

The analysis of the nucleotide sequence of the L1 gene of MfoiPV1 using the PAVE L1 Taxonomy Tool (https://pave.niaid.nih.gov/#analyze/l1_taxonomy_tool, accessed on 8 February 2021) suggests that the virus is a new type within the genus *Dyonupapillomavirus* with 67.61% identity to the most closely related type AmPV3. Currently, three members of the genus *Dyonupapillomavirus* are known, all from Carnivora hosts, namely AmPV3 from *Ailuropoda melanoleuca* (giant panda), ZcPV1 from *Zalophus californianus* (California sea lion), and LwPV3 from *Leptonychotes weddellii* (Weddell seal).

**Figure 3:**
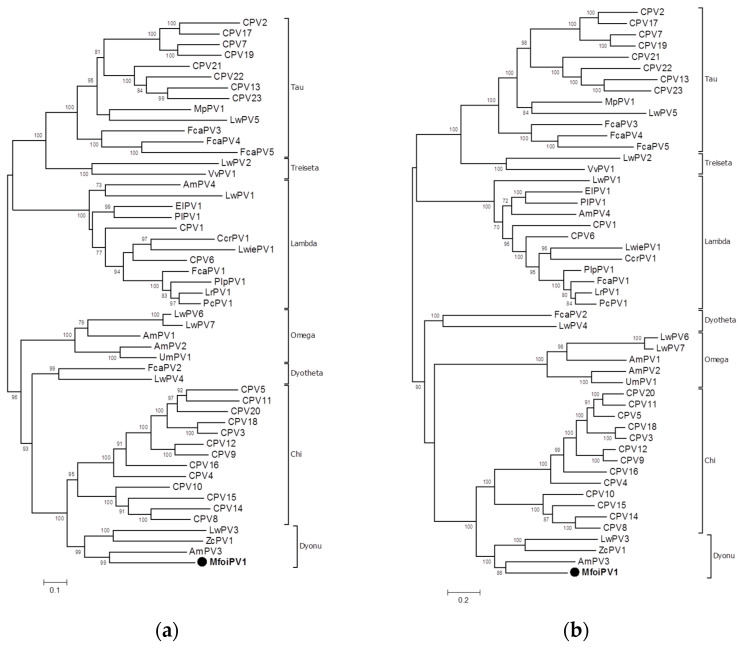
Phylogenetic trees of PVs from Carnivora hosts. Genome from this study is written in bold and marked with a solid circle. Statistical support for the phylogenetic tree was evaluated via bootstrapping based on 1000 repetitions. Bootstrap values lower than 70 are not shown. The trees are drawn to scale. Genera are indicated by Greek letters, according to PaVE database (http://pave.niaid.nih.gov, accessed on 8 February 2021). (**a**) Maximum likelihood (ML) tree of the L1 gene nucleotide sequences; and (**b**) maximum likelihood (ML) tree of the concatenated E1E2L2L1 amino acid sequences.

The analysis of multiple alignments revealed that the L1 gene of MfoiPV1 has 65.47%, 62.76%, and 60.05% nucleotide identity with other members of the *Dyonupapillomavirus* genus, the AmPv3, ZcPV1, and LwPV3, respectively. Phylogenetic analysis of the nucleotide sequences of the L1 gene (Figure 3a) and the concatenated amino acid sequences of the E1E2L2L1 genes (Figure 3b) confirmed the closest relationship of MfoiPV1 to the AmPv3, ZcPV1 and LwPV3 types, as a strongly supported cluster of these sequences was revealed. The classification of papillomaviruses is based on the criterion that the nucleotide sequences of their L1 genes should have a nucleotide identity of more than 60% within the same genus, 60–70% within the same species and more than 90% within the same type [3,49]. Following these classification criteria, MfoiPV1 is proposed to be classified as a new PV type and also assigned to a new species within the genus *Dyonupapillomavirus*.

## 3. Discussion

The biology of the free-ranging stone marten has been studied extensively, but little is known about pathogens including PV in this species. Although wildlife health surveillance in Slovenia dates back several decades, few stone marten carcasses have been submitted for postmortem examination and few diagnoses have been confirmed. The main factor contributing to the low number of diagnoses is the advanced decomposition of many carcasses. The most common diagnoses were trauma and distemper.

In the case presented in this paper, a solitary skin tumor was observed in a stone marten carcass found in poor body condition. The histopathological examination of the skin tumor revealed elements characteristic of an acrochordon. Acrochordons or fibroepithelial polyps are benign skin tumors with a clinical presentation that varies from single to multiple nodules, exophytic to pedunculated, and lesions can occur in dermal and epidermal tissues [50]. These benign tumors have been reported in rare studies in dogs [50,51], but are a common condition in humans [52]. Study results in humans have shown an association between human papillomavirus (HPV) and acrochordons, suggesting a viral cause of acrochordons [53,54]. The etiology of acrochordons in dogs is unknown, but it is suggested that traumatic lesions and infections may promote their growth [50,55]. In the case from this study, it is difficult to say whether PV or trauma was the cause of tumor growth. The histopathological findings are not typical of fibropapillomas; nevertheless, we cannot exclude a PV as the cause of acrochordon in this stone marten.

To determine the complete genome of PV detected in a tumor of the studied stone marten, the tumor tissue sample was subjected to WGS using NGS technology. After complete genome assembly, the genome was annotated and compared with the PV types available in the PaVE database using the L1 taxonomy tool, and a new PV type belonging to the *Dyonupapillomavirus* genome was proposed. The proposed new stone marten (*Martes foina*) PV type was named MfoiPV1 and its genome was characterized in further detail.

The genome organization analysis of MfoiPV1 and its genome characteristics at the nucleotide and amino acid levels were compared with other members of the *Dyonupapillomavirus* genus and revealed similar genome organization and main amino acid and nucleotide sequence characteristics. Phylogenetic analyzes revealed the closest relationship of MfoiPV1 to the AmPv3, ZcPV1, and LwPV3 types. Nucleotide sequence analysis of the L1 gene showed less than 66% nucleotide sequence identity of MfoiPV1 compared to the aforementioned most similar PV types. According to PV classification criteria, this is consistent with the identification of a new PV type; therefore, we propose MfoiPV1 as a new type within the genus *Dyonupapillomavirus*, which could also belong to a new species.

According to the PaVE database, 50 papillomaviruses have been identified to date in various wild and domestic carnivore hosts. Carnivora host-associated papillomaviruses belong to the genera *Chi*-, *Lambda*-, *Tau*-, *Omega*-, *Treiseta*-, *Dyotheta*- and *Dyonupapillomavirus*. The most studied PVs of Carnivora hosts are from domestic dogs (types CPV1—CPV23), belonging to the genera *Chi*-, *Lambda*- and *Taupapillomavirus* [8,13,26]. Within the mustelids, PVs have only been reported in southern sea otters infected with *Enhydra lutris* papillomavirus 1 (ElPV1), which causes oral papillomatosis [56], and in fecal material from a ferret infected with *Mustela putorius* papillomavirus 1 (MpPV1) [57]. These viruses belong to the *Tau*- (MpPV1) and *Lamdapillomavirus* (ElPV1) genera. Until this study, three members of the genus *Dyonupapillomavirus* were known, all from Carnivora hosts, namely AmPV3 from giant panda [39], ZcPV1 from California sea lion [58] and LwPV3 from Weddell seal [59]. ZcPV1 was the only PV type of the genus *Dyonupapillomavirus* associated with papillomavirus lesions, whereas no association with clinical disease was found for either AmPV3 from nasopharyngeal secretions of the giant panda or LwPV3 from vaginal swabs of the Weddell seal.

## 4. Materials and Methods

### 4.1. Case Description and Sample Collection

An adult male stone marten was found dead by a local hunter the hunting area of Idrija in western Slovenia in August 2019 and sent to Veterinary Faculty (University of Ljubljana, Ljubljana, Slovenia) for necropsy. Permission from Ethics Committee/Welfare Authority was not required, as all samples were collected postmortem. Samples for histopathology were fixed in 10% buffered formalin, processed, embedded in paraffin, sectioned, and stained with hematoxylin and eosin, according to standard protocols. For bacteriology, culture from tissue samples was performed on Columbia agar (Oxoid LTD, Basingstoke, Hampshire, UK) supplemented with 5% sheep blood and incubated aerobically and anaerobically at 37 °C for 72 h. The isolate was analyzed by matrix-assisted laser desorption—ionization time of flight—mass spectrometry MALDI-TOF—MS (Bruker Daltronik GmbH, Bremen, Germany) according to the manufacturer’s instructions.

Bacteriological examination of the samples revealed a mixed bacterial flora. Histopathological examination of the lung and kidney tissue revealed verminous pneumonia and chronic pyelitis. Based on the gross and histopathological findings and bacterial culture results, a diagnosis of multifactorial disease was made.

A sample from the skin tumor was collected for a papillomavirus detection with PCR and stored at −70 °C until analysis.

### 4.2. Papillomavirus PCR

The skin tumor sample was suspended in RPMI medium 1640 (Thermo Fisher Scientific, Carlsbad, CA, USA). The suspension was homogenized and centrifuged at 2000× *g* for 10 min, and the supernatant was employed for nucleic acid extraction using DNeasy Blood & Tissue Kit according to the manufacturer’s instructions (Qiagen, Germany). PCR with a combination of primers (CanPVf: 5′-CTTCCTGAWCCTAAYMAKTTTGC-3′, FAP64: 5′-CCWATATCWVHCATITCICCATC-3′) amplifying a 383 bp-long fragment of the L1 gene was used to detect PVs [60,61]. PCR products were subjected to electrophoresis in a 1.8% agarose gel.

### 4.3. Whole-Genome Sequencing, Bioinformatics Analysis of NGS Data and Genome Annotation

Illumina (Illumina, USA) next-generation sequencing (NGS) technology was used for whole-genome sequencing. The extracted DNA was used for the preparation of the NGS library. Paired-end sequencing (2 × 150 bp) was performed on the NovaSeq 6000 Illumina platform. Library preparation and sequencing were performed by Novogene.

SPAdes software v.3.10.0 [62] was used for the de novo assembly of reads. The assembled contigs were subjected to a BlastX search using Diamond [63] to determine those representing PV sequences. BlastX results were analyzed using MEGAN6 [64] for taxonomic assignment of reads using the Lowest Common Ancestor (LCA) algorithm.

The Geneious Prime software suite v. 2019.1.3 (Biomatters Ltd., Auckland, New Zealand) was used for further downstream bioinformatic analyses of the assembled MfoiPV1 genome. To eliminate assembly errors and finalize the sequence of the circular MfoiPV1 genome, all sequenced reads were mapped against the assembled genome using the Geneious reference mapper (Geneious Prime software suite v. 2019.1.3, Biomatters Ltd., Auckland, New Zealand). The MfoiPV1 genome sequence was aligned with the PV genomes of *Dyonupapillomavirus* genus from GenBank, namely the AmPV3 (MF327537), the ZcPV1 (HQ293213) and the LwPV3 (MG571093) using MAFFT [65]. Open reading frames (ORFs) were predicted with the Geneious ORF Finder, and the assembled genome was annotated based on multiple alignments relative to the three *Dyonu*PV genomes. cNLS Mapper (http://nls-mapper.iab.keio.ac.jp/cgi-bin/NLS_Mapper_form.cgi, accessed on 10 March 2021) [66] was used to predict the NLS. Sequence was deposited in GenBank under the accession number (MW841296).

### 4.4. Phylogenetic Analysis

All available PV genomes of the Carnivora host origin (*n* = 50) in the PaVE database [15] were retrieved from the database and used for phylogenetic comparisons. These PV genomes belong to *Tau*-, *Treiseta-*, *Lambda-*, *Dyotheta-*, *Omega-*, *Chi-* and *Dyonu-* PV genera. From all these annotated genome sequences, the E1, E2, L1 and L2 gene sequences were extracted and translated into amino acid sequences using the Geneious Prime software suite v. 2019.1.3 (Biomatters Ltd., Auckland, New Zealand). Multiple alignments of the concatenated E1, E2, L2 and L1 amino acid sequences were generated using MAFFT [65]. Multiple alignments of the L1 gene nucleotide sequences were also created using MAFFT with the translation align option. Using the multiple alignments mentioned above, phylogenetic trees were constructed by maximum likelihood method with the IQ-TREE software [67] and visualized in MEGA7 [68]. The best-fitting substitution model determined according to BIC scores was LG+F+I+G4 and the GTR+F+I+G4 for the E1, E2, L2, L1 tree and L1 tree, respectively. Statistical support for the phylogenetic trees was evaluated by bootstrapping based on 1000 repetitions.

## 5. Conclusions

The histological presentation of the skin tumor in the present case was unusual for a typical PV-induced papilloma; nevertheless, the tumor contained DNA sequences belonging to papillomavirus. Using the NGS technology, the complete genome of a novel PV type belonging to the *Dyonupapillomavirus* genus (MfoiPV1) was determined. Whether this virus causes skin lesions in stone martens requires further investigation. This finding expands our knowledge of PV diversity in wildlife and may help to improve our understanding of the evolution of PV.

## Figures and Tables

**Table 1:** Positions of ORFs in the MfoiPV1 genome and predicted amino acid (aa) and nucleotide (nt) features.

ORF			Predicted aa Features		
Name	Position in Genome	Aa Length	Name	Sequence	Aa Start Position in ORF
E6	1–456	152	Zinc-binding domains	CXXCX(29)CXXC	25, 98
E7	416–712	99	RB protein binding domain	LXCXE; in MFoiPV1: LWCDE	23
			Zink-binding domains	CXXCX(29)CXXC	51
E1	702–2603	634	ATP-dependent helicase motif	GXXXXGK[TS]; in MFoiPV1: GPPNTGKS	461
			Cyclin interaction motifs	RXL	56, 113, 278, 398, 446, 561
E2	2545–4116	524	/		
E4	3116–3874	253	/		
L2	4268–5761	498	/		
L1	5777–7273	499	Bipartite nuclear localization signal	RKFLAQSSATARPVKRRAPP	467
			Predicted nt features		
Name			Name	Sequence	Nt Start Position in Genome
LCR			E2 binding sites	ACCGNNNNCGGT;	7714, 7638, 7578, 7545, 7336
LCR			TATA-like box	TATA[AT]A[AT]; in MFoiPV1: TATATATA	7748
LCR			SP1 binding site	GGCGGG	7772
E2			NF1 binding site	CGGAAA	3767
E1, E2, L2, L1			Polyadenylation signal sites	ATAAA	1434, 3038, 4359, 6443, 6858

## Data Availability

The data presented in this study are available on request from the corresponding author.
